# Dual-energy CT with virtual monoenergetic images to improve the visualization of pancreatic supplying arteries: the normal anatomy and variations

**DOI:** 10.1186/s13244-022-01157-z

**Published:** 2022-02-04

**Authors:** Hong-wei Liang, Yang Zhou, Zhi-wei Zhang, Gao-wu Yan, Si-lin Du, Xiao-hui Zhang, Xin-you Li, Fa-jin Lv, Qiao Zheng, Yong-mei Li

**Affiliations:** 1 Department of Radiology, The First Affiliated Hospital of Chongqing Medical University, No. 1 Youyi Road, Yuzhong District, Chongqing, 400016 China; 2 Department of Radiology, Suining Central Hospital, Suining, 629000 China

**Keywords:** Pancreatic supplying arteries, Dual-energy CT, Virtual monoenergetic images

## Abstract

**Background:**

Pancreatic ductal adenocarcinoma (PDAC) remains a malignancy with poor prognosis, appropriate surgical resection and neoadjuvant therapy depend on the accurate identification of pancreatic supplying arteries. We aim to evaluate the ability of monoenergetic images (MEI [+]) of dual-energy CT (DECT) to improve the visualization of pancreatic supplying arteries compared to conventional polyenergetic images (PEI) and investigate the implications of vascular variation in pancreatic surgery and transarterial interventions.

**Results:**

One hundred patients without pancreatic diseases underwent DECT examinations were retrospectively enrolled in this study. The signal-to-noise ratio (SNR) and contrast-to-noise ratio (CNR) at 40-keV MEI (+) were significantly higher than those of PEI (*p* < 0.05). All subjective MEI (+) scores were significantly higher than those of PEI (*p* < 0.05). The visualization rates were significantly higher for posterior superior pancreaticoduodenal artery (PSPDA), anterior and posterior inferior pancreaticoduodenal artery (AIPDA, PIPDA), anterior and posterior pancreaticoduodenal arcade (APAC, PPAC), transverse and caudal pancreatic artery (TPA, PCA) at 40-keV MEI (+) than those of PEI (*p* < 0.05). However, there were no significant differences for visualizing anterior superior pancreaticoduodenal artery (ASPDA), inferior pancreaticoduodenal artery (IPDA), dorsal and magnificent pancreatic artery (DPA, MPA) between 40-keV MEI (+) and PEI (*p* > 0.05). Four types of variations were observed in the origin of DPA and three to five types in the origin of PSPDA, AIPDA and PIPDA.

**Conclusions:**

40-keV MEI (+) of DECT improves the visualization and objective and subjective image quality of pancreatic supplying arteries compared to PEI. Pancreatic supplying arteries have great variations, which has important implications for preoperative planning of technically challenging surgeries and transarterial interventions.

## Key points

- 40-keV MEI (+) of DECT improves visualization of pancreatic supplying arteries compared to PEI.
- 40-keV MEI (+) of DECT improves objective and subjective image quality of pancreatic supplying arteries compared to PEI.
- The normal anatomy and variations in pancreatic supplying arteries have important implications for technically challenging surgeries and transarterial interventions.

## Introduction

Pancreatic ductal adenocarcinoma (PDAC) is the fourth leading cause of cancer deaths in the USA, with an overall survival rate of 6–7% [1, 2]. Complete surgical resection (R0) is the only curative option to obtain long-term survival [3, 4]. For unresectable pancreatic cancer, transarterial chemotherapies have been applied as a neoadjuvant therapy [5–7]. Appropriate surgical resections and local arterial interventions depend on a comprehensive understanding of pancreatic supplying arteries and their variations.

Multidetector computed tomography (MDCT) with thin-section imaging has been used to assess the peripancreatic arterial anatomy [8, 11]. However, the polychromatic X-ray beam used in a conventional MDCT is more susceptible to beam hardening artifacts [9]. Due to the relatively low resolution of conventional MDCT, this technique permits the major visceral arteries such as the celiac artery (CA), common hepatic artery (CHA) and superior mesenteric artery (SMA) to be identified but does not provide sufficient information on the anatomy of small pancreatic supplying arteries [8]. The recently introduced third generation dual-energy CT (DECT) is the latest tube-based DECT solution, which allows for the reconstruction of monoenergetic images (MEI [+]) by using the noise-optimized monoenergetic algorithm [11]. MEI (+) approximates the image obtained by monoenergetic X-rays, in which iodine attenuation increases as the energy approximates the iodine k-edge (33.2 keV), so as to acquire higher vessel contrast [3, 13]. Nagayama and Beer have demonstrated the better visualization at 40-keV MEI (+) in the peripancreatic arteries, but they had only evaluated the major visceral arteries [3, 14].

The pancreas receives multiple arterial supplies, leading to a wide variation in its arterial blood distribution, may alter the surgical plan and cause complications in transarterial interventions [7]. It is, therefore, crucial to obtain accurate information on both normal anatomy and variations in pancreatic supplying arteries, but sometimes MDCT cannot provide sufficient information on the pancreatic blood distribution. In such cases, DECT can provide a detailed and accurate assessment. However, the performance of DECT in demonstration of pancreatic supplying arteries and their variations have been scantly evaluated.

The aim of this study is to evaluate the ability of 40-keV MEI (+) of DECT to improve the visualization of pancreatic supplying arteries compared to PEI and to discuss the implications of the normal anatomy and variations in pancreatic supplying arteries for pancreatic surgery and interventional therapy.

## Materials and methods

This retrospective study received institutional review board approval; the requirement for written informed consent was waived.

### Patients

**Fig. 1:**
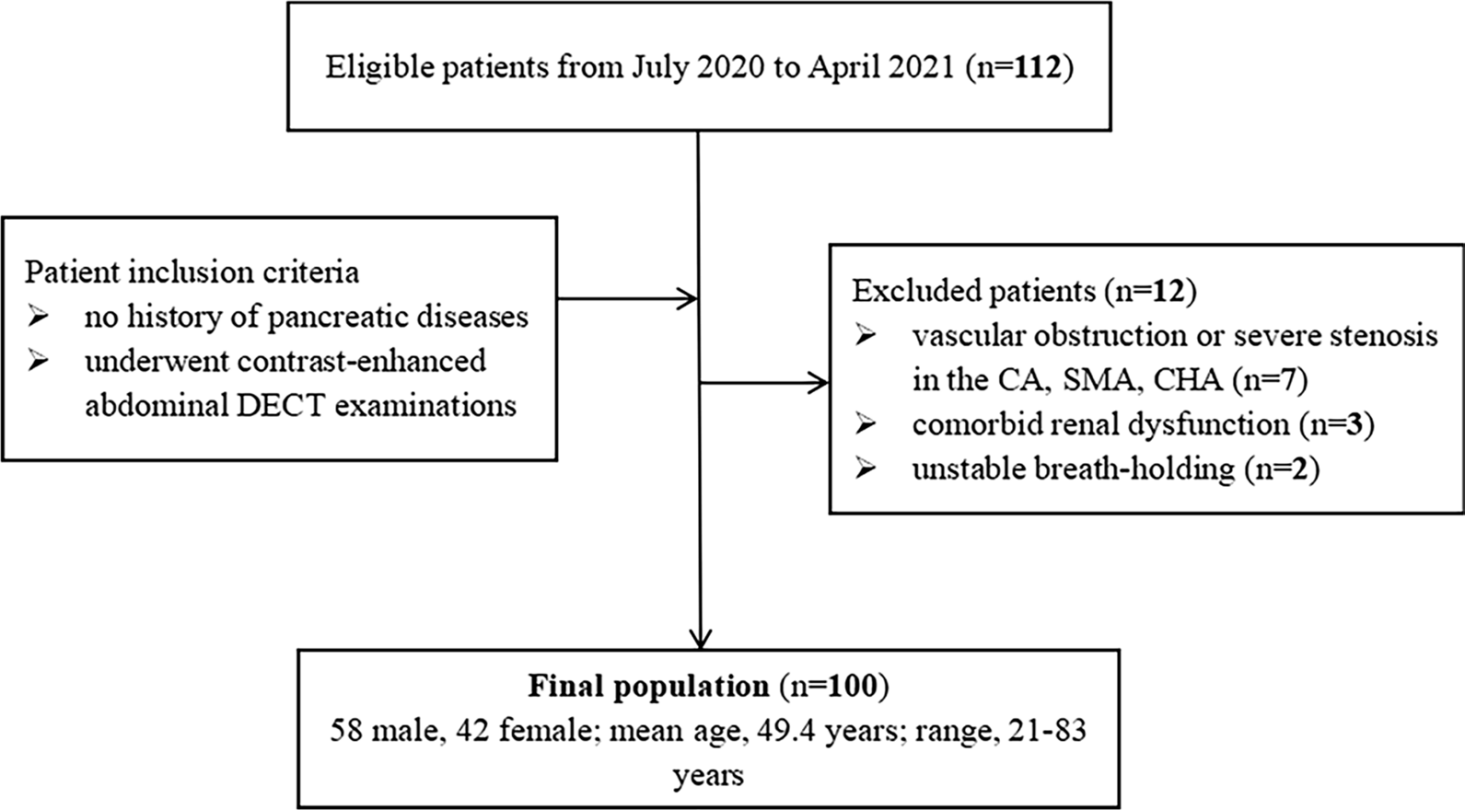
Flowchart of the included and excluded patients

We identified 112 consecutive patients without pancreatic diseases who accepted contrast-enhanced abdominal DECT examinations on a dual-source DECT scanner (SOMATOM Force, Siemens Healthineers) between July 2020 and April 2021. Overall, 12 of 112 patients were excluded for one of the following reasons: vascular obstruction or severe stenosis in the CA, SMA, CHA (n = 7), comorbid renal dysfunction resulted in a reduction in contrast medium protocol (n = 3), and unstable breath-holding (n = 2), for a final cohort size of 100 patients (Fig. 1).

### CT examinations

Image data were acquired on a DECT scanner in dual-energy mode through two X-ray tubes with different kV tube voltages (tube A, 100 kV; tube B, Sn 150 kV), using a tin filter for the high-voltage tube. Settings for both scanners were as follows: collimation 128 × 0.6 mm; rotation time 0.5 s; pitch 0.6; reference tube current time product for the A tube, 260 mAs. Automatic exposure control (CAREDose4D) was used in all scans. Images were obtained in a craniocaudal direction from the hepatic dome to bilateral anterior superior iliac spine.

A non-ionic contrast agent (Ultravist 370, Bayer Healthcare; or Iopamiro 370, Bracco Healthcare) was injected at a dose of 1.2 mL/kg and at a flow rate of 3.5–5 mL/s through a peripheral vein of the forearm, followed by a 40-mL saline flush at the same injection rate. Arterial phase scanning was started with a delay of 10 s after a trigger threshold in the abdominal aorta (100 HU) was reached, portal venous phase scanning was carried out 30 s after the end of the arterial phase.

### Image reconstruction

Reconstructed CT image data were post-processed on a syngovia workstation (syngo.via, version VB20A; Siemens Healthineers). Standard linear-blended images were reconstructed by applying a blending factor of 0.6 (M_0.6; 60% of the 100 kV and 40% of the Sn150 KV spectrum) to represent a conventional 120 kV impression. MEI (+) was reconstructed at 40 keV using the novel noise-optimized monoenergetic reconstruction algorithm. Axial, sagittal, and coronal images were reformatted with a thickness of 1 mm, an increment of 1 mm. Maximum intensity projection (MIP) and volume rendering (VR) were created for both MEI (+) and PEI by a radiologist.

### Quantitative analysis

All these series were analyzed on a syngovia workstation. Objective quantification was performed by a radiologist with 6 years of experience in abdominal imaging. Attenuation of different areas was measured in Hounsfield units (HU), the CT attenuations of pancreatic parenchyma (HU_pancreas_, averaged from normal pancreas parenchyma), peripancreatic arteries (HU_artery_, averaged from gastroduodenal artery [GDA], ASPDA, and DPA), and retroperitoneal fat (HU_fat_) were measured by placing circular regions of interest (ROIs). Image noise was quantified as the standard deviation (SD) of HU_fat_. For pancreatic attenuation, care was taken to exclude the visible pancreatic duct, vessels, and artifacts. For vessel analysis, firstly, observation and location of pancreatic arteries were performed on the coronal MIP images. If considered visible, the axial images were magnified three times and then used for quantitative analysis, the ROI was placed in the middle of the vessel cavity as far as possible. The above measurements were performed twice and then averaged to confirm data consistency. The quantitative image quality of each object (pancreatic parenchyma, peripancreatic arteries) was calculated with Eqs. (3, 14):

$$\begin{aligned} & {\text{CNR}}_{{{\text{object}}}} = ({\text{HU}}_{{{\text{object}}}} - {\text{HU}}_{{{\text{pancreas}}}} ) \, /{\text{SD}}_{{{\text{fat}}}} \\ & {\text{SNR}}_{{{\text{object}}}} = {\text{HU}}_{{{\text{object}}}} /{\text{SD}}_{{{\text{fat}}}} \\ \end{aligned}$$

### Qualitative analysis

Two radiologists with 6 and 10 years of experience of abdominal imaging, respectively, independently reviewed the axial and reformatted images. They were blinded to any patient clinical information and reconstruction parameters. PEI and MEI (+) obtained from the artery phases were separately assessed in random order. Based on the findings of previous reports [10, 11, 15], the ASPDA, PSPDA, IPDA AIPDA, PIPDA, DPA, MPA, TPA, CPA, APAC and PPAC were identified, and the frequency of visualization and variations in these arteries were recorded. Five-point scales were used to assess visualization of arteries: 5 (excellent, the origin of the vessel is identified and its anatomic course is clearly traced; vessel strengthened obviously), 4 (good, the origin of the vessel is identified, and its anatomic course is traced with reasonable certainty; vessel strengthened well), 3 (average, the origin of the vessel is identified, but only the proximal part of its anatomic course is traced; the edge vessel is not sharp or fuzzy), 2 (suboptimal, the origin of the vessel is identified; the vessels are slender), 1 (poor, the origin of the vessel is not identified; the vessels are not clear). The frequency of visualization and variation in the arteries were assessed by two radiologists, and the final decisions were reached by consensus.

### Statistical analysis

Statistical analysis was performed using SPSS version 26.0 (IBM Cor, 2013) and GraphPad Prism V5. The Kolmogorov–Smirnov test was applied to assess the normality of data distribution. The continuous variables were expressed as means ± SD. Chi-square test was applied to assess differences in visualization rate of pancreatic supplying arteries between MEI (+) and PEI. An independent t test was performed to compare the quantitative parameters. A Mann–Whitney U test was performed to compare the qualitative parameters. The inter-reader agreement for qualitative analysis was assessed using Cohen’s kappa (> 0.81, excellent; 0.61–0.80, substantial; 0.41–0.60, moderate; 0.21–0.40, fair; and < 0.20, poor agreement). Differences of *p* < 0.05 were considered statistically significant.

## Results

### Objective image parameters

**Fig. 2:**
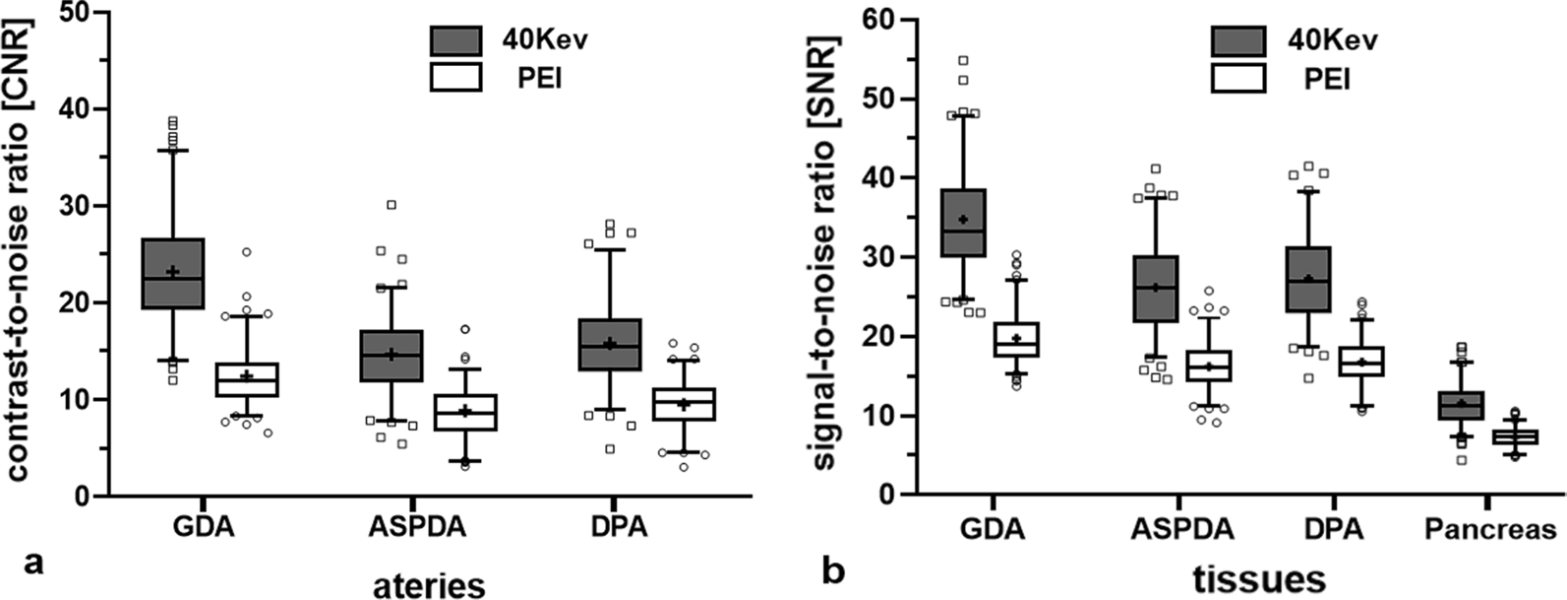
Box-and-whisker plots of the differences in CNR **(a)** and SNR **(b)** between 40-keV MEI (+) and PEI. The box indicates the 25- and 75-quartile; the horizontal line indicates the median and the cross indicates the mean. Whiskers show the 5 and 95 percentiles; outliers are indicated by squares and circles

40-keV MEI (+) significantly improved the CNR and SNR of the GDA, ASPDA, and DPA compared with the PEI (*p* < 0.05). For all vessels analyses, compared with PEI, the CNR and SNR of the GDA obtained at 40-keV MEI (+) increased by 60.03% and 50.76%, respectively (*p* < 0.001). The CNR and SNR of the ASPDA obtained at 40-keV MEI (+) increased by 41.60% and 38.66%, respectively (*p* < 0.05). The CNR and SNR of the DPA obtained at 40-keV MEI (+) increased by 42.43% and 39.20%, respectively (*p* < 0.001) (Fig. 2).

For pancreatic parenchyma, the SNR obtained at 40-keV MEI (+) increased by 58.14% compared with PEI (*p* < 0.001). The image noise at 40-keV MEI (+) was significantly higher than that of PEI (*p* < 0.001). Full results of the quantitative analysis are shown in Table 1.

**Table 1:** Quantitative analysis

	40-keV MEI(+)	PEI	*p*
Image noise	22.49 ± 3.24	14.32 ± 1.49	< 0.001
*CNR*			
GDA	23.22 ± 5.95	12.43 ± 3.09	< 0.001
ASPDA	14.67 ± 4.31	8.88 ± 2.83	0.011
DPA	15.81 ± 4.66	9.49 ± 2.63	< 0.001
*SNR*			
GDA	34.78 ± 7.04	19.74 ± 3.46	< 0.001
ASPDA	26.22 ± 5.54	16.18 ± 3.08	< 0.001
DPA	27.31 ± 5.82	16.74 ± 3.07	< 0.001
Pancreatic parenchyma	11.56 ± 2.78	7.31 ± 1.31	< 0.001

Data shown are mean ± standard deviation

CNR*,* contrast-to-noise ratio; SNR, signal-to-noise ratio; GDA, gastroduodenal artery; ASPDA, anterior superior pancreaticoduodenal artery; DPA, dorsal pancreatic artery

### Subjective image quality

Compared with PEI, 40-keV MEI (+) provided significantly higher subjective scores for all the pancreatic supplying arteries (*p* < 0.05) (Table 2). There was moderate to substantial inter-reader agreement for 40-keV MEI (+) (kappa = 0.56–0.79) and PEI (kappa = 0.55–0.77), respectively.

**Table 2:** Qualitative analysis

	ASPDA	PSPDA	IPDA	AIPDA	PIPDA	DPA	MPA	TPA	PCA
40-keV MEI (+)	4.19 ± 0.82*	3.58 ± 0.97*	4.18 ± 0.69*	3.42 ± 0.79*	2.97 ± 0.77*	4.10 ± 0.79*	3.57 ± 0.73*	3.44 ± 0.77*	2.88 ± 0.54*
PEI	3.34 ± 0.85	2.99 ± 0.80	3.71 ± 0.79	2.86 ± 0.69	2.50 ± 0.69	3.45 ± 0.71	2.89 ± 0.61	2.69 ± 0.74	2.35 ± 0.49
Kappa	0.74	0.64	0.65	0.73	0.62	0.72	0.67	0.75	0.71

Data shown are mean ± standard deviation

*Scores for 40-keV MEI (+) > scores for PEI (*p* < 0.05)

ASPDA, anterior superior pancreaticoduodenal artery; PSPDA, posterior superior pancreaticoduodenal artery; IPDA, inferior pancreaticoduodenal artery; AIPDA, anterior inferior pancreaticoduodenal artery; PIPDA, posterior inferior pancreaticoduodenal artery; DPA, dorsal pancreatic artery; MPA, magnificent pancreatic artery; TPA, transverse pancreatic artery; PCA, caudal pancreatic artery

### The frequency of visualization of vessels

The display rates were significantly higher for PSPDA, AIPDA, PIPDA, APAC, PPAC, TPA, and PCA at 40-keV MEI (+) than in PEI (96% vs 88%; 96% vs 87%; 93% vs 86%; 60% vs 45%; 53% vs 38%; 84% vs 72%; 64% vs 49%; *p* < 0.05). However, there were no significant differences for visualizing ASPDA, IPDA, DPA, and MPA between 40-keV MEI (+) and PEI (100% vs 99%; 51% vs 48%; 92% vs 86%; 87% vs 82%; *p* > 0.05) (Table 3).

**Table 3:** The frequency of visualization of arteries

Artery	40-keV MEI (+) (%)	PEI (%)	*P*
ASPDA	100	99	0.316
PSPDA	96	88	0.037*
IPDA	51	48	0.671
AIPDA	96	87	0.022*
PIPDA	93	86	0.046*
APAC	60	45	0.034*
PPAC	53	38	0.033*
DPA	92	86	0.175
MPA	87	82	0.329
TPA	84	72	0.041*
PCA	64	49	0.032*

*40-keV MEI (+) > PEI in the frequency of visualization of arteries (*p* < 0.05)

ASPDA, anterior superior pancreaticoduodenal artery; PSPDA, posterior superior pancreaticoduodenal artery; IPDA, inferior pancreaticoduodenal artery; AIPDA, anterior inferior pancreaticoduodenal artery; PIPDA, posterior inferior pancreaticoduodenal artery; APAC, anterior pancreaticoduodenal arcade; PPAC, posterior pancreaticoduodenal arcade; DPA, dorsal pancreatic artery; MPA, magnificent pancreatic artery; TPA, transverse pancreatic artery; PCA, caudal pancreatic artery

### The normal anatomy and variations in vessels at 40-keV MEI (+)

#### Superior pancreaticoduodenal artery (ASPDA and PSPDA)

**Fig. 3:**
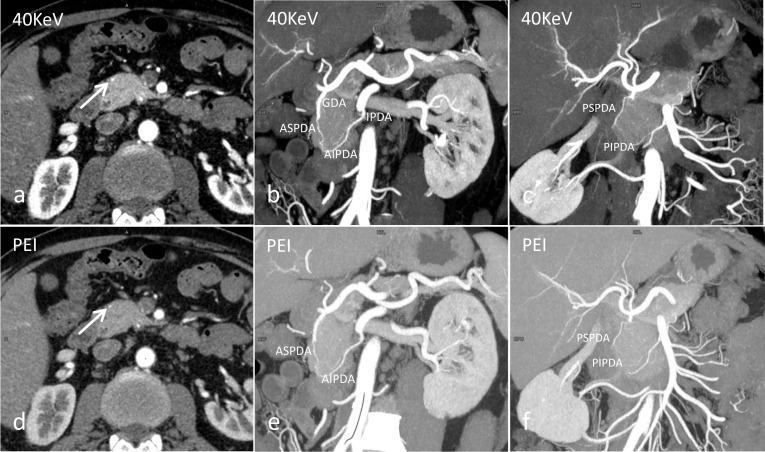
The upper **(a–c)** and lower **(d–f)** rows show the 40-keV MEI (+) and PEI images, respectively. **a** The axial image shows that the anterior superior pancreaticoduodenal artery (ASPDA) runs along the anterior and lateral surface of the pancreatic head (arrow). **b** Coronal reformatted image shows the ASPDA originating from gastroduodenal artery (GDA), and anastomosing inferiorly with anterior inferior pancreaticoduodenal artery (AIPDA) to form the standard anterior pancreaticoduodenal arcade. **c** Coronal reformatted image shows the posterior superior pancreaticoduodenal artery (PSPDA) and the posterior inferior pancreaticoduodenal artery (PIPDA). The visualization is better on the 40-keV MEI (+), compared to the PEI

The ASPDA originated as minor terminal branch of the GDA in all the cases, in 63 cases, anastomoses between the ASPDA and AIPDA were apparent, forming a standard single anterior arcade (n = 60) (Fig. 3b), two arcades (n = 10), and an anastomotic branch with the DPA (prepancreatic Kirk arcade) (n = 13).

**Fig. 4:**
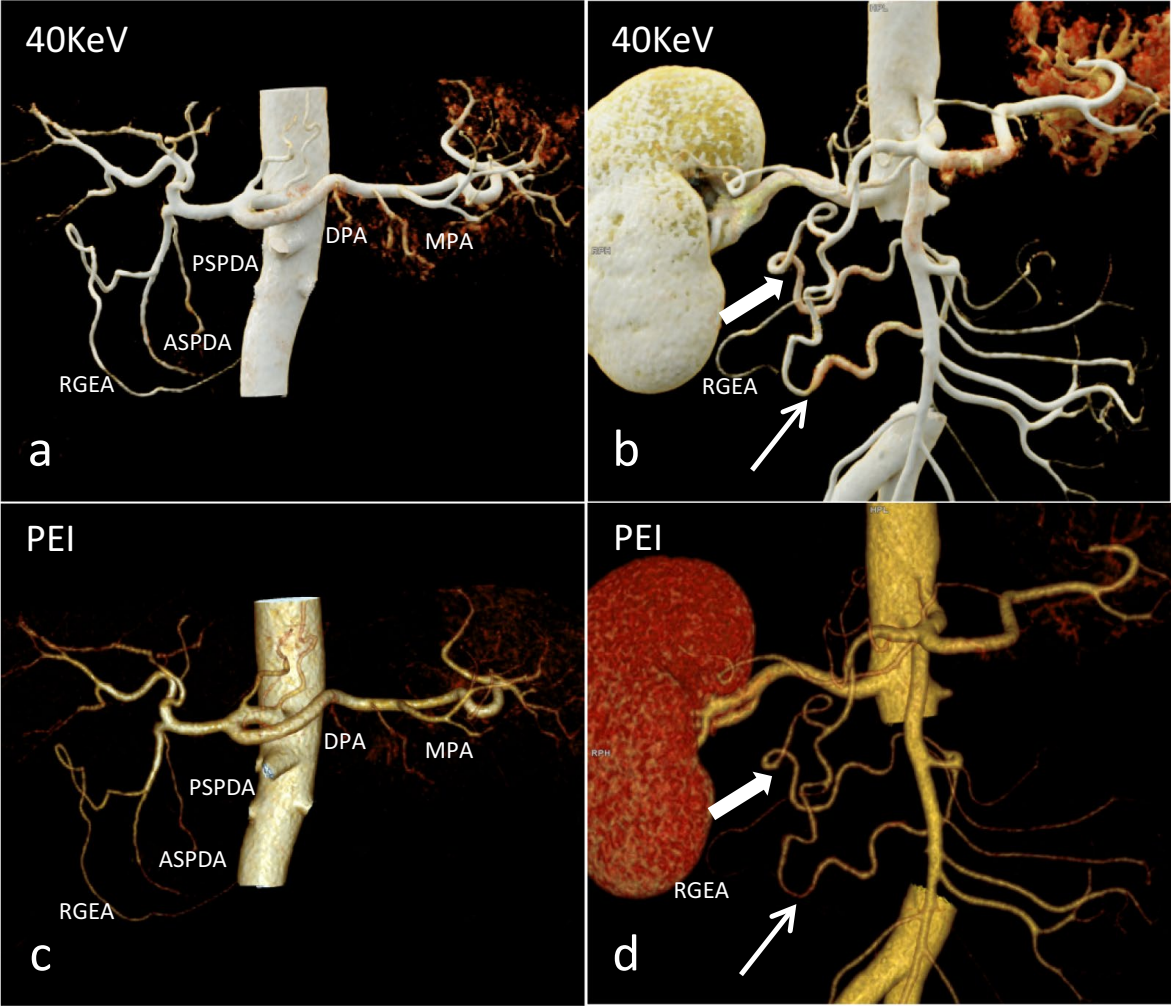
The upper **(a, b)** and lower **(c, d)** rows show the 40-keV MEI (+) and PEI images, respectively. **a** Volume Rendering Technology (VRT) of contrast-enhanced CT angiography shows the anterior superior pancreaticoduodenal artery (ASPDA), posterior superior pancreaticoduodenal artery (PSPDA), dorsal pancreatic artery (DPA) and magnificent pancreatic artery (MPA). **b** VRT shows standard anterior (thin arrow) and posterior (thick arrow) pancreaticoduodenal arcade. The visualization is better on the 40-keV MEI (+), compared to the PEI. RGEA = Right gastroepiploic artery

Three types of variations were observed in the origin of PSPDA. The PSPDA arose from (a) GDA (n = 92; Fig. 3c); (b) aberrant right hepatic artery (a/r RHA) from SMA (n = 2); (c) PHA (n = 2). The collateral branches of PSPDA were visualized: retroduodenal arteries (n = 28); in 59 cases, the anastomoses with the PIPDA were appreciable, with a standard single posterior arcade (n = 53) (Fig. 4b), two arcades (n = 6) and an anastomotic branch with the DPA (retropancreatic arcade) (n = 8); and in 4 cases, it had both the prepancreatic and retropancreatic arcade.

### Inferior pancreaticoduodenal artery (IPDA, AIPDA, PIPDA)

**Fig. 5:**
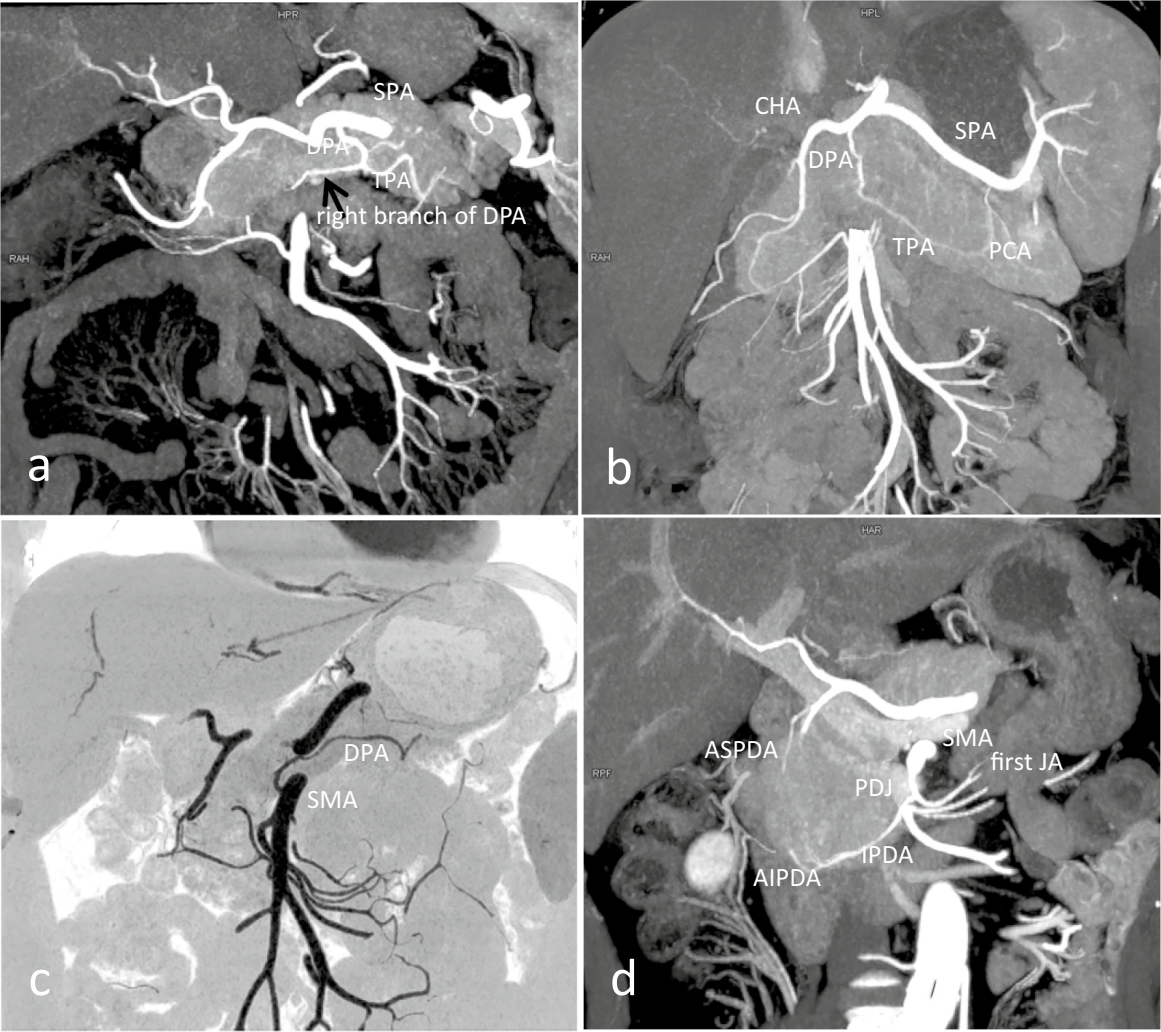
The normal anatomy and variations in pancreatic supplying arteries. **a** MIP shows the dorsal pancreatic artery (DPA) originating from splenic artery (SPA), bifurcates into the right branch and the transverse pancreatic artery (TPA), which forms an inverted “T” pattern branching. **b** The DPA originates from the common hepatic artery (CHA), bifurcates into the TPA, and then anastomoses with caudal pancreatic artery (PCA). **c** Minimum intensity projection (MinIP) shows the DPA originating from the superior mesenteric artery (SMA). **d** MIP shows the inferior pancreaticoduodenal artery (IPDA) originating from the first jejunal artery (JA) to form the pancreatico-duodeno-jejunal (PDJ) trunk

The IPDA predominantly originated from SMA and bifurcated into the AIPDA and PIPDA (n = 35). Other origins of the IPDA were from a common trunk with the first jejunal artery (JA) forming a pancreatico-duodeno-jejunal (PDJ) trunk (n = 15; Fig. 5d) and from the middle colon artery (n = 1).

In the cases which did not have an IPDA, four to five types of variations were observed in the origin of AIPDA and PIPDA (Table 4). The AIPDA arose from (a) SMA (n = 27); (b) DPA (n = 4); (c) first JA (n = 12); (d) a/r RHA (n = 1); (e) CHA (n = 1). And the PIPDA arose from (a) SMA (n = 24); (b) DPA (n = 7); (c) first or second JA (n = 5); (d) a/r RHA (n = 2).

**Table 4:** The major origin of IPDA, AIPDA, and PIPDA

Origin of IPDA	Variations in AIPDA and PIPDA origin	% (n)
SMA	AIPDA and PIPDA from IPDA bifurcation	35% (35/100)
From a common trunk (PDJ) with first jejunal artery (JA)	AIPDA and PIPDA from IPDA bifurcation	14% (14/100)
middle colon artery	AIPDA and PIPDA from IPDA bifurcation	1% (1/100)
Absent IPDA	Separate origin of AIPDA and PIPDA from SMA	22% (22/100)
Absent IPDA	AIPDA from first JA and PIPDA from SMA	5% (5/100)
Absent IPDA	PIPDA from DPA and AIPDA from SMA	5% (5/100)
Absent IPDA	AIPDA from SMA and PIPDA absent	2% (2/100)
Absent IPDA	AIPDA from SMA and PIPDA from a/r RHA from SMA	2% (2/100)

IPDA, inferior pancreaticoduodenal artery; AIPDA, anterior inferior pancreaticoduodenal artery; PIPDA, posterior inferior pancreaticoduodenal artery; DPA, dorsal pancreatic artery; SMA, superior mesenteric artery; a/rRCHA, aberrant right hepatic artery; JA, jejunal artery; PDJ, pancreaticoduodenojejunal trunk

### Dorsal pancreatic artery (DPA)

The DPA supplied the body of the pancreas, which had the greatest variability. The DPA originated from (a) splenic artery (SPA) (n = 62; Fig. 5a); (b) SMA (n = 14; Fig. 5c); (c) CHA (n = 11; Fig. 5b); (d) CA (n = 5). At the lower edge of the pancreas, DPA bifurcated into the right branch and left terminal branches, which formed an inverted “T” pattern branching (n = 25). The left branch corresponded to the TPA, while the right branch constituted the prepancreatic Kirk arcade with the ASPDA (n = 13) and the retropancreatic arcade with the PSPDA (n = 8).

### Transverse pancreatic artery (TPA)

The TPA arose from (a) DPA and represented its left branch (n = 71; Fig. 5a); (b) the left branch of ASPDA (n = 8); (c) PDJ trunk (n = 3). There were anastomoses between the TPA and MPA (n = 35) and the first JA (n = 2).

## Discussion

This study demonstrated that 40-keV MEI (+) of DECT provided significantly better objective and subjective image quality in depicting pancreatic supplying arteries relative to conventional PEI. The visualization rates were significantly higher for PSPDA, AIPDA, PIPDA, APAC, PPAC, TPA, and PCA at 40-keV MEI (+) than in PEI. Furthermore, pancreatic supplying arteries have great variabilities, which have important implications for preoperative planning of technically challenging surgeries and transarterial interventions.

In previous studies, some authors have indicated that MEI (+) reconstructions can yield improvement in the visualization of the major visceral arteries [3, 12, 14]. Nagayama and Beer have demonstrated the higher SNR and CNR at 40-keV MEI (+) in peripancreatic arteries (CA, CHA, SMA) [3, 14]. These studies favored low keV MEI (+) in terms of subjective and objective image parameters. However, the performance of DECT in demonstration of small pancreatic supplying arteries and their variations had been scantly investigated. In this study, we evaluated the ability of 40-keV MEI (+) of DECT to improve visualization of pancreatic supplying arteries compared with PEI and analyzed their normal anatomy and variations, using a much larger patient cohort.

The visualization of the enhanced vessels is mainly dependent on the image noise and the degree of vascular enhancement. However, reducing noise requires to increase radiation dose; improving the degree of vascular enhancement requires to increase the contrast dose and flow rate. However, previous study suggested that the fast injection rate increases the risk of extravasation and hyperosmolarity of contrast material [16]. CNR is the main objective parameter of vessel contrast. DECT MEI (+) can improve the degree of vascular enhancement without increasing the dose of radiation and contrast agent, so as to improve the CNR of enhanced vessels [17]. In our study, the CNR of GDA, ASPDA, and DPA at 40-keV MEI (+) increased by 60.03%, 41.60%, and 42.43%, respectively, compared with PEI.

Image noise is another factor affecting the visualization of the pancreatic supplying arteries. The objective image noise of 40-keV MEI (+) is higher than that of PEI in our study, which in accordance with recent investigations that have shown the increased noise for lower energy levels [12, 14]. However, compared with standard monoenergetic algorithm, a novel noise-optimized monoenergetic algorithm had been introduced to overcome this limitation in the third generation DECT. This technique performs a spatial frequency-based recombination that reduces the image noise of lower energies and improves image contrast at higher energies to obtain the best image quality [3, 14]. In addition, although image noise is often used as an image quality indicator, the consideration for the SNR level is also necessary. Increased SNR could necessarily guarantee improved image quality if the absolute noise level is clinically acceptable [12]. In our study, the SNR of pancreatic parenchyma, GDA, ASPDA, and DPA in 40-keV MEI (+) increased by 58.14%, 50.76%, 38.66%, and 39.20%, respectively, compared with PEI. Moreover, an advanced modeled iterative reconstruction was applied for DECT to suppress the image noise and improve image quality [18, 19]. All these reasons may contribute to the better image quality at 40-keV MEI (+) than PEI.

Improving the visualization of pancreatic supplying arteries is important for some planning limited surgery, such as duodenum-preserving resection of the pancreatic head [20]. In order to perform surgical resection of the pancreatic head safely, it is crucial to clearly depict the anatomy of the pancreaticoduodenal arcades [8]. In addition, familiarization with the normal anatomy and variations in pancreatic supplying arteries is important in planning transarterial interventions. Tanaka et al. demonstrated that the optimal drug distribution in arterial infusion chemotherapy for advanced PDAC was related with proper placement of the catheter [21]. The catheter placement in arterial infusion depends largely on the precise knowledge about the blood distribution and variations because the arteries supply to each region of the pancreas is different [21]. In our study, except the ASPDA, IPDA, DPA and MPA, the visualization rates of pancreatic supplying arteries at 40-keV MEI (+) were significantly higher than that of PEI. One potential explanation may be that the ASPDA, IPDA, DPA and MPA have a relatively thick luminal diameter and high visualization rate at the conventional PEI, so the visualization rate at 40-keV MEI (+) was not significantly improved [11, 22, 23].

Pancreas has complex arterial supply, and the anatomy of pancreatic supplying arteries is highly variable, both in origin and distribution [24]. In our study, the display rates were higher for ASPDA and PSPDA at 40-keV MEI (+) than in PEI (100% vs 99%; 96% vs 88%). And high variability was observed in their origin. At 40-keV MEI (+), ASPDA arose from GDA in 100% and PSPDA in 95.83% cases, like the previously reported data (95–100% and 92.5–96.2%, respectively) [10, 15]. In the remaining cases, the PSPDA originated from PHA and a/r RHA from SMA in four cases. Generally, blood supply of the superior head of the pancreas is always from the GDA and CA, and Sakuhara et al. suggested that CT during arteriography is not required to confirm the supplying artery in these regions [7]. However, in our study, PSPDA originated from a/r RHA from SMA in two cases. It is crucial to be familiar with such arterial patterns before super-selective transarterial interventions.

The IPDA was present in 51% cases at 40-keV MEI (+) in our study, which was higher than the PEI (48%). At 40-keV MEI (+), IPDA originated from SMA in 35 cases, and PDJ trunk in 15 cases. Other origin of IPDA was the middle colon artery in one case. Knowledge of this rare variation is essential to avoid potential complications during pancreatic surgical and transarterial interventions. The occurrence of AIPDA and PIPDA has been reported by most of the previous studies with an overall incidence of 96–100% [10, 11]. In our study, these were found in 96% and 93% cases at 40-keV MEI (+), respectively. In the cases which did not have an IPDA, the other sources of origin of AIPDA in our study were SMA in 27 cases, first JA in 12 cases, DPA, a/r RHA and CHA in 6 cases. On the other hand, the PIPDA arose from SMA in 24 cases, DPA in 7 cases, first or second JA and a/r RHA in 7 cases. The DPA has the greatest variability of all the arteries supplying the pancreas. In our study, the DPA was present in 92% cases at 40-keV MEI (+), which was higher than the PEI (86%). Doppman et al. suggested that the DPA predominantly originates from the SPA and to supply the body of the pancreas [25]. In our study, the DPA has been found to arise from SPA in 62 cases, which was mostly in agreement with theirs, other origins were SMA in 14 cases, CHA in 11 cases and CA in 5 cases. This result indicated that the body of the pancreas is supplied by the SMA when the DPA originates from the SMA.

This study has several limitations. Firstly, image analysis was based on conventional PEI and 40-keV MEI (+) reconstructions. Other MEI (+), such as 50-keV or 60-keV, were not assessed since previous studies have shown 40-keV was the optimal MEI (+). Secondly, the pancreatic supplying arteries did not have anatomic reference standard in our study. However, we believe that our artery identification was accurate, and it was confirmed that the findings essentially accord with those of previous anatomic and radiological reports. Finally, we did not analyze the influence of pancreatic diseases on pancreatic supplying arteries. Therefore, further investigations should be performed on the relationship between pancreatic diseases and pancreatic supplying arteries.

## Conclusion

In conclusion, 40-keV MEI (+) of DECT substantially improves the visualization and objective and subjective image quality of pancreatic supplying arteries compared to conventional PEI. Accurate preoperative identification with the normal anatomy and variations in pancreatic supplying arteries has important clinical significance for pancreatic surgery and transarterial interventions.

## Data Availability

The datasets used and/or analyzed during the current study are available from the corresponding author on reasonable request.

## Abbreviations

a/r RHA: Aberrant right hepatic artery
AIPDA: Anterior inferior pancreaticoduodenal artery
APAC: Anterior pancreaticoduodenal arcade
ASPDA: Anterior superior pancreaticoduodenal artery
CA: Celiac artery
CHA: Common hepatic artery
CNR: Contrast-to-noise ratio
DECT: Dual-energy CT
DPA: Dorsal pancreatic artery
GDA: Gastroduodenal artery
IPDA: Inferior pancreaticoduodenal artery
JA: Jejunal artery
MEI (+): Monoenergetic images
MPA: Magnificent pancreatic artery
PCA: Caudal pancreatic artery
PDJ: Pancreatico-duodeno-jejunal trunk
PEI: Polyenergetic images
PIPDA: Posterior inferior pancreaticoduodenal artery
PPAC: Posterior pancreaticoduodenal arcade
PSPDA: Posterior superior pancreaticoduodenal artery
SMA: Superior mesenteric artery
SNR: Signal-to-noise ratio
TPA: Transverse pancreatic artery
